# Evaluation of enteric methane production in dairy cows fed *Acacia mearnsii*

**DOI:** 10.5455/javar.2025.l962

**Published:** 2025-09-22

**Authors:** Lindokuhle C. Mhlongo, Ignatius V. Nsahlai

**Affiliations:** 1 Department of Animal Science, University of the Free State, Bloemfontein, South Africa; 2 Department of Animal and Poultry Sciences, University of KwaZulu–Natal, Scottsville, South Africa

**Keywords:** *Acacia mearnsii*, CH_4_, greenhouse gas, rumen–headspace gases, tannins

## Abstract

**Objectives:**

We investigated the effect of different incorporations of *Acacia mearnsii* forage (AM) in maize silage or *A. mearnsii* tannin extract (AME) in pellets on dairy rumen CH_4_.

**Materials and Methods:**

Using a completely randomized design per experiment, 24 crossbred Holstein–Friesian and Jersey dairy cows per experiment were divided into groups (*n* = 6 cows per experiment). Dairy cows were fed pellets with 0% (0PEL), 0.75% (0.75PEL), 1.5% (1.5PEL), or 3.0% (3PEL) of AME (Experiment 1). Furthermore, dairy cows were fed 0% (0AM), 5% (5AM), 15% (15AM), or 25% (25AM) of AM in maize silage (Experiment 2). Data sampling period (21 days) of ruminal CH_4_ and nitrogen (N_2_), carbon dioxide (CO_2_), and hydrogen (H_2_) gases (% vol) was conducted after the adaptation period (14 days) for each experiment.

**Results:**

Enteric CH_4_ was not affected by AME inclusion, but AM inclusions affected CH_4,_ except for CH_4_ (% vol per cow per day). The inclusions of 25AM decreased CH_4_ per nutrient intake (kg/day), such as dry matter (DM), organic matter (OM), crude protein, acid detergent fiber (ADF), and neutral detergent fiber (NDF). In addition, there was a linear and quadratic AM inclusion effect on CH_4_ per intake of nutrients, including DM, NDF, ADF, and OM.

**Conclusion:**

Enteric CH_4_ was not affected by AME but was decreased by AM in dairy cows.

## Introduction

Methane (CH_4_) gas is produced during the digestion of fiber, a component of carbohydrate fermented in the rumen by rumen microbes [1]. This gas results from pyruvate breakdown into acetate during fiber digestion, where more hydrogen (H_2_) molecules are produced for methanogens to utilize to produce CH_4_ [2]. This gas contributes to global warming, and a large amount of agricultural CH_4_ emissions comes from ruminant production [3]. In addition, the production of CH_4_ also typically wastes feed since ruminants lose 2% of their energy intake from producing this gas [4]. Therefore, to decrease ruminant production costs and safeguard the environment, ruminant CH_4_ emissions must be minimized using natural strategies.

Due to their capacity to reduce ruminant CH_4_ generation, condensed tannins (CTs) have received scientific attention as a solution for this challenge [5,6]. Certain feeds naturally contain CTs, which bind nutrients to reduce CH_4_ in ruminants and are a potential solution for this challenge. CT-rich feeds lower CH_4_ by decreasing acetate, the acetate–propionate ratio, methanogens, and the population of protozoa, which all have an indirect role in the synthesis of CH_4_ [7]. Despite this, some studies have produced contradictory results, indicating that CT sources do not affect CH_4_ emission [8].

*Acacia mearnsii* is a tannin source that can be used as an *A*. *mearnsii* tannin extract (AME) or an *A*. *mearnsii* forage (AM) to depress CH_4_ emissions in cows. However, *A. mearnsii* has mostly been utilized to depress CH_4_ in ruminants in the form of AME to lower CH_4_ generation in ruminants [3]. There have been reports of AME failure in lowering CH_4_ in ruminants [9], which necessitates the assessment of AM or AME inclusions on CH_4_. Most of the literature available on the use of AM or AME to depress CH₄ in ruminants relies on *in vitro* studies, a poor predictor of *in vivo* CH₄. There is a need to expand the assessment of AM or AME under on*-*farm conditions to improve the understanding of CH_4_ response *in vivo*.

Research on the AM or AME inclusions under on-farm conditions can also improve the comprehension of how tannin sources affect CH_4_. The emission of CH_4_ depends on the dietary components of the basal diet fed to ruminants. Investigations into the inclusion of AM or AME in different basal diets on CH_4_ are needed to identify AM or AME inclusions that depress CH_4_ for a given type of basal diet. We investigated the effect of different incorporations of AM in maize silage or AME in pellets on dairy rumen CH_4_. We hypothesized that increasing AM or AME incorporation would decrease enteric CH_4_ in dairy cows.

## Materials and Methods

### Ethical approval

The Animal Research Ethics Committees of KwaZulu-Natal Department of Agriculture and Rural Development [ref: 12/11/1/15 (2387JD)] and University of KwaZulu-Natal (ref: AREC/00003470/2021) approved this research.

### Study site

The study site for this research was a commercial dairy farm, Springfontein dairy farm located in Kokstad, Kwa–Zulu–Natal province, South Africa (coordinates, 270°W, 30°15^�^52^��^S, 29°10^�^15^��^E S and altitude, 1450 m). 

### Acacia mearnsii-enriched pellets 

A total of 24 cows were divided into four treatment groups (*n* = 6 dairy cows per treatment group) by a complete randomization design. The treatment groups were control 0% (0PEL), 0.75% (0.75PEL), 1.5% (1.5PEL), or 3.0% (3PEL) addition of powdered *Acacia* *mearnsii* (AME) in a pelleted mixed feed (Tables 1 and 2). Before the 21-day data collection period, cows were adapted to treatments for 14 days [10]. Using animal spray paint for identification, cows in each treatment group were tagged with the diets they were assigned. The intakes of 0PEL, 0.75PEL, 1.5PEL, and 3PEL were the difference between feed left and feed fed (as is). During milking, the study site’s herd was given a 15% commercial concentrate of crude protein (CP) (AFGRI, Pietermaritzburg, South Africa), which was used as 0PEL. The manufacturer withheld information regarding the ingredient composition of 0PEL. Tannin purity of AME was 68% and was determined using the butanol–hydrochloric acid method (Mimosa, Pietermaritzburg, South Africa).

Crossbred Holstein–Friesian and Jersey cows being mechanically milked [60*-*point rotational milking machine (E100, De Laval, Pinetown, South Africa)] and with 199.5 ± 5.12 days in milk (mean ± SD) were used in this study. On the farm, there were no precise lactation numbers available for each cow. Every morning and afternoon milking session, each cow received 2 kg (as*-*fed) of their allocated pellets using the milking unit feeders.

Dairy cows were fed *ad libitum* soybean and maize silages in a fenced camp overnight. During the day, dairy cows grazed perennial ryegrass and white clover pasture between morning (5:00 am) and afternoon (14:00) milking sessions. Before and after milking, cows were screened for mastitis.

### Acacia mearnsii-enriched maize silage

Using complete randomization, 24 crossbred Jersey and Holstein–Friesian cows of 211.46 ± 16.41 days in milk (days in milk ± SD) were assigned to 0% (0AM), 5% (5AM), 15% (15AM), and 25% (25AM) (as*-*fed) AM in maize silage diets (Table 3), whereby each treatment was assigned six cows. Since the farm lacked precise statistics regarding the herd’s lactation numbers, parity was not used in the study design. Before the 21*-*day data collection period, cows were adapted to treatments for 14 days [10]. Using animal spray paint for identification, cows in each treatment group were tagged with the diets they were assigned. The intakes of 0AM, 5AM, 15AM, and 25AM were measured weekly from the difference between feed left and feed given (as is).

**Table 1.** Composition of experimental pellets.

Items (kg)	3PEL	1.5PEL	0.75PEL
Crushed maize	498	498	498
Hominy chop	275	275	275
Bran	100	100	100
Molasses liquid	50	50	50
Soya oil cake	30	30	30
Urea	15	15	15
Lime powder	12.5	12.5	12.5
Salt	10	10	10
Monocalcium phosphate 21	5	5	5
Mineral and vitamin premix	5	5	5
Condensed tannin extract powder	44	22	11

Weighed fresh corn silage gathered from the pit each day was combined with various AM inclusions, transferred into labelled bags, mixed, and fed to treatment groups daily. AM (twigs and leaves) was ground with a wood chipper (Tomcat Wood Chipper, Worcester, South Africa) and dehydrated by sunlight under shelter (7 days) pending use.

Following afternoon milking (14:00 pm), cows were fed treatments (2 kg, as fed for 2 h) in lockable, open*-*air wooden individual pens. Every pen had a gate constructed with barbed wire and iron standards and fresh water (20 l) in plastic containers. Each pen was constructed with slatted poles (51 cm spacing), into which plastic feeders were affixed so that each cow could fit their head to get feed.

Dairy cows were fed lucerne and veld hay (*ad libitum*) in the overnight camp. During the day, dairy cows grazed white clover and perennial ryegrass. Dairy cows were given a custom feed concentrate (15% CP; AFGRI, Pietermaritzburg, South Africa) during milking and were teat-dipped every quarter for mastitis pre- and post-milking*.*

### Methane determination

Every week, after milking in the morning, cows were gently immobilized in the headlock gate. Hair was clipped, and the left paralumbar fossa area was cleaned (F10). The ruminal gases were collected at the *paralumbar fossa* region of the cows by injecting a needle (14 G needle and 60 ml syringe with a three*-way* stopper). Sampled gases (CH₄, N₂, CO₂, and H₂, % vol) were injected into the portable multiple gas analyzer (SKZ–1050D, China) for headspace gas (% vol) determination.

### Feed sampling and proximate analysis

Feed was sampled weekly by diagonally sampling each pasture paddock and sampling the top, middle, and bottom sections of each feed bale. Thereafter, feed samples were separately refrigerated in labeled feed bags while pending analysis.

Feed samples were oven*-*dried (60°C for 72 h), ground (2 mm sieve), packed in labeled zip-lock bags, and sent to the Cedara Analytical Laboratory (Hilton, South Africa) for proximate analysis using AOAC methods [11]. The nitrogen analyzer (Leco Truspec, LECO, Pretoria, South Africa) was used to quantify nitrogen content, and the nitrogen content (ID 968.06) × 6.25 equaled the CP content estimate (Leco FP200, LECO). Dry matter (DM) in an air-forced oven and ash content resulted from combustion in a muffle furnace. Furthermore, ANKOM 200 fiber analyzers (ANKOM Technology, Macedon, New York) were used for neutral detergent fiber (NDF) and acid detergent fiber (ADF) determination [12]. The 810 Fat Analyzer determined ether extract content (Soxhlet Buchi, Flawil, Switzerland). The AM samples contained in a medium-sized zip-lock bag were sent to the MIMOSA extract company (NTE House, Redlands Estate, Pietermaritzburg, South Africa) for tannin content determination [13].

**Table 2.** Chemical composition of the basal feeds and experimental pellets.

Items (gm/kg)	DM	Ash	EE	ADF	NDF	CP
Maize silage	964.4	39.0	28.5	220.0	396.0	65.0
Perennial ryegrass + White clover	941.0	150.0	18.5	222.0	356.0	217.0
^#^PEL	916.3	64.0	26.5	48.9	163.8	145.3
0PEL	980.0	88.5	22.5	110.1	280.7	150.0
Soya silage	573.0	193.0	–	281.0	345.0	11.5

ADF, acid detergent fiber; CP, crude protein; DM, dry matter; NDF, neutral detergent fiber*;. ^#^Chemical composition of experimental pellets without inclusion of tannins ; 0PEL, custom concentrate pellets which were used as the control.

**Table 3.** Chemical composition of experimental feeds.

Feeds	Chemical composition (gm/kg)							
	DM	Ash	EE	ADF	NDF	CP	ADIN	CT
Lucerne hay	967.3	108	28.7	304.2	406.4	119.6	9.3	–
AM	879.7	44.7	31.8	658.3	753.3	157	16.5	31
0AM	964.6	56.5	25.8	339.8	607.4	81	6.6	–
Veld hay	940.4	63.3	14.8	522.2	844.1	69.1	5.4	–
Ryegrass + white clover	964.5	121.8	18.5	293.1	536.5	219.1	12.6	–
5AM	966.8	201	20.1	289.6	476.8	67.2	–	1.56
15AM	969.2	49.4	24.4	37.73	615.1	75.4	–	4.65
25AM	964	46	29.3	454.7	641.3	97.9	–	7.75

0AM, 0% AM inclusion in corn silage; 5AM, 5% AM inclusion in CS; ADF, acid detergent fiber; ADIN, acid detergent insoluble nitrogen; CP, crude protein, CT, condensed tannins; DM, dry matter; EE; ether extract; NDF; neutral detergent fiber.

### Statistical analyses and calculations

The DM intake was determined by multiplying the feed intake of treatments (as-fed) by their corresponding DM content. CH₄ per nutrient intake of nutrients was determined by multiplying the intake of each of the above-mentioned nutrients by its corresponding CH₄ volume measured (% vol). The data were analyzed using statistical analysis software (SAS, 9.4), general linear model procedure using the following model: Yi = m + ai + ei, where Yi is the response variable, ai is the treatment incorporation effect, and ei is the error term. The Tukey test was used to separate the means. The differences among the least square means were tested by Tukey’s test for statistical significance of *p* < 0.05 by diets on the dependent variables. Furthermore, linear and quadratic effects were ascertained using the orthogonal contrast statement.

## Results

The effect of AME incorporation in pellets on the gases and CH_4_ in dairy cows is demonstrated in Table 4. The effect of AME incorporation in pellets on CH_4_ and other gases was absent (*p *> 0.05). Furthermore, there was the absence of the linear and quadratic trend effects of AME incorporations on CH_4_ and other gases and CH_4_ (*p *> 0.05).

The effect of AM incorporation in maize silage diets on CH_4_ in dairy cows is demonstrated in Table 5. The effect of AM incorporation in maize silage and their linear and quadratic trend effects on CH_4_ %, CO_2_, and N_2_ (% vol) were absent (*p *> 0.05). H_2_ had 0% vol content in the rumen headspace. Different AM inclusions affected CH_4_ per kg intake of DM, CP, OM, NDF, and ADF (*p* < 0.05). Linear and quadratic trend effects of AM incorporation on CH_4_ per kg intake of DM, CP, OM, NDF, and ADF were observed (*p *< 0.05). However, CH_4_ per kg nutrient intake of DM, CP, OM, NDF, and ADF were similar at 5%–15% AM incorporations but decreased at the 25% AM incorporations compared to the control.

## Discussion

### Effect of AME inclusions on methane emissions in dairy cows

AMEs are used to limit CH_4_ in livestock, but their use is preliminary due to their inconsistency in CH_4_ emissions. The inconsistency of AME inclusions necessitates their investigation to identify inclusions that limit CH_4_. Our results showed that including AME in pellets of up to 3% does not affect CH_4_ production or nutrient intake. Our hypothesis was rejected, as it assumed that the incremental inclusions of *A*. *mearnsii* tannins in pellets decrease CH₄ emissions.

Demonstration of previous results shows that 0%, 0.75%, 1.5%, and 2.25% inclusions of AME* *do not affect CH_4_ emissions [14]. This is consistent with other tannin extract types, such as mimosa and quebracho, that do not affect CH_4_ emissions and nutrient intake at inclusion rates of up to 1% [15] or 2% [16] in the diet. This suggests that the inclusions of AME used in the current study were insufficient to control CH_4_ emissions. Supplementation of field grass with 0.12% and 0.18% tannin decreased CH_4_ emissions and increased DM intake with suppression of CP digestibility [17,18]. Another finding was that tannin extract decreased CH_4_ with a decrease in nutrient intake and digestibility [19]. Therefore, AME inclusions used in the present study may have been due to failure to elicit nutrient digestibility suppression as well as their intakes.

In some instances, AME inclusions of up to 20% decrease CH_4_ with a decrease in gas production [20]. It is possible that the decrease in CH_4_ was not realized due to the failure to suppress gas production, which contains substrates for CH_4_ production, CO_2_, and H_2_ [1]. However, inclusions of 0%, 0.5%, 1%, and 1.5% AME decreased CH_4_ emissions with suppression of rumen microbes [21]. Decrement of methanogenesis relies on the depression of nutrient intake and digestibility. Our study was limited to grouping cows according to their milk emission yield, a factor with a positive relationship with CH_4_ production. Therefore, further studies should investigate the effect of AME in cows of different milk yields.

### Effect of AM inclusion on methane emissions in dairy cows

Reducing CH_4_ emissions in dairy cows using nonconventional feeds such as *Acacia mearnsii *forage (AM) potentiates a solution to controlling climate change. Our study showed that increased inclusions of AM in maize silage decreased CH_4_ emissions as a proportion of DM, OM, CP, ADF, and NDF intakes. The decline of CH_4_ gas as a proportion of the above*-*mentioned nutrients correlated with decreased nutrient intake. Therefore, the 25% inclusion of AM in maize silage depresses CH_4_ gas as a proportion of nutrient intake, but with nutrient intake dependence. We tested the hypothesis that the increment of AM in maize silage would decrease CH_4_ production. This partially agreed with our hypothesis because CH_4_ gas declined with inclusion of AM, but was equally refuted as the enteric CH_4_ as a nutrient intake proportion was not affected by AM incorporation in maize silage.

**Table 4.** The effect of dietary inclusions of *Acacia mearnsii* tannin extract in pellets on enteric gases in dairy cows (experiment 1).

Items	0PEL	0.75PEL	1.5PEL	3PEL	SEM	Significance		
						GLM	Linear	Quadratic
Nutrient intake (kg/day, DM)								
DMI	3.67	3.63	3.67	3.67	0.02	0.413	0.66	0.33
OMI	3.43	3.4	3.43	3.43	0.02	0.413	0.66	0.33
CPI	0.53	0.53	0.53	0.53	0.003	0.413	0.66	0.33
NDFI	0.60	0.59	0.60	0.60	0.003	0.413	0.66	0.33
ADFI	0.18	0.18	0.18	0.18	0.0008	0.413	0.66	0.33
CH_4_ (% vol) and CH_4_ per nutrient intake (kg/day)								
CH_4_	19.76	19.95	19.83	20.38	0.42	0.7229	0.3574	0.6729
CH_4_/DMI	0.72	0.72	0.73	0.75	0.03	0.5362	0.2231	0.4458
CH_4_/OMI	0.68	0.68	0.68	0.70	0.01	0.5670	0.2451	0.4532
CH_4_/CPI	0.11	0.11	0.11	0.11	0.0027	0.4670	0.2221	0.3588
CH_4_/NDFI	0.12	0.12	0.12	0.12	0.12	0.7600	0.7352	0.4521
CH_4_/ADFI	0.04	0.04	0.04	0.04	0.0019	0.8559	0.6990	1.000
Other gases (% vol)								
H_2_	0	0	0	0	–	–	–	–
N_2_	30.73	29.33	30.74	29.23	0.78	0.3529	0.3896	0.9448
CO_2_	47.08	48.95	46.11	48.52	0.98	0.1836	0.7407	0.7858

Significant difference, *p* < 0.05; CP, crude protein; DM, dry matter; GLM, general linear model; NDF, neutral detergent; OM, organic matter; SEM, standard error of means.

**Table 5.** Effect of different dietary inclusions of *Acacia mearnsii* forage in pellets on enteric gases in dairy cows (experiment 2).

Items	0AM	5AM	15AM	25AM	SEM	Significances		
						GLM	Linear	Quadratic
Nutrient intake (kg/day)								
DMI	4.90^a^	4.47^a^	4.36^a^	3.13^b^	0.15	<0.0001	<0.0001	0.0078
CPI	0.40^a^	0.39^a^	0.41^a^	0.30^b^	0.01	<0.0001	<0.0001	0.0006
OMI	4.62^a^	4.28^a^	4.11^a^	2.93^b^	0.14	<0.0001	<0.0001	0.0032
ADFI	1.65^a^	1.62^a^	1.68^a^	1.22^b^	0.06	<0.0001	<0.0001	0.0008
NDFI	2.95^a^	2.80^a^	2.74^a^	1.99^b^	0.09	<0.0001	<0.0001	0.0014
CH_4_ (% vol) and CH_4_ per nutrient intake (kg/day)								
CH_4_	23.07	23.98	23.02	22.07	1.00	0.5423	0.7620	0.4140
CH_4_/DMI	1.13^a^	1.07^a^	1.00^a^	0.68^b^	0.06	<0.0001	0.0001	0.0470
CH_4_/CPI	0.09^a^	0.09^a^	0.09^a^	0.07^b^	0.01	0.0002	0.0256	0.0200
CH_4_/OMI	1.07^a^	1.02^a^	0.94^a^	0.64^b^	0.05	<0.0001	0.0001	0.0358
CH_4_/ADFI	0.38^a^	0.39^a^	0.39^a^	0.27^b^	0.02	<0.0001	0.0161	0.0161
CH_4_/NDFI	0.68^a^	0.67^a^	0.63^a^	0.44^b^	0.04	<0.0001	0.0009	0.0287
Other enteric gases (% vol)								
H_2_	0	0	0	0	–	–	–	–
N_2_	32.80	32.92	36.37	35.59	1.65	0.2510	0.1501	0.8325
CO_2_	39.81	37.86	36.12	36.35	1.58	0.2986	0.0793	0.4445

Significant difference, *p* < 0.05; ADF, acid detergent fiber; CP, crude protein; DM, dry matter; GLM, general linear model; NDF, neutral detergent fiber; OM, organic matter; SEM, standard error of means.

Similarly, previous studies showed that up to 20% AM inclusion decreases CH_4_ gas production [22]. Furthermore, AM tannins decrease CH_4_ emissions in association with nutrient intake [10]. Conversely, lipid*-*encapsulated AM decreased CH_4_ without negatively affecting nutrient intake [23]. Depression of CH_4_ in association with that of nutrient intake attributes a lack of encapsulation of AM in the present study [24]. The lack of tannin encapsulation can cause tannins to limit nutrient intake through their astringent taste. Thus, AM tannins may have depressed substrate availability for CH_4_-producing microbes to produce CH_4_.

CH_4_ production depends on nutrient digestibility. Alternatively, our results for AM demonstrate that AM inclusions decreased nutrient digestibility. AM leaf meal decreases CH_4_ gas and DM degradability in the rumen [25]. DM degradability depression by AM lowers protozoa populations, which contribute to CH_4_ emissions [25].

Our study was limited by not using the exact AM inclusions and basal diet that were previously tested *in vitro* [26]. This would have quantified how certain inclusions of AM tannins tested *in vitro* affect CH_4_ emissions *in vivo*. This limitation necessitates the need for future studies to investigate AM inclusions that have been previously investigated for their effect on digestibility and rumen microbial populations to ascertain the AM inclusions associated with the depression of CH_4_ without performance decrease [27]. 

## Conclusion

Feeding dairy cows varying AME inclusions in pellets did not affect CH_4_ production. However, feeding dairy cows varying incorporations of AM decreased CH_4_ per intake of DM, CP, OM, NDF, and ADF with linear and quadratic trend effects. Different inclusions of AME need to be assessed for cows of different CH_4_ emission levels. Furthermore, *in vivo* studies are necessary to confirm these findings using milk yield and previously tested inclusions of AME under* in vitro* conditions.

## List of abbreviations

ADF, acid detergent fiber; AM, *Acacia mearnsii* forage; AME, *Acacia mearnsii* tannin extract; CH4, methane gas; CO_2_, carbon dioxide gas; CP, crude protein; CT, condensed tannins; DM, dry matter content; H_2_, hydrogen; N_2_, nitrogen gas; NDF, neutral detergent fiber; OM, organic matter; PEL, pelleted concentrate; SAS, statistical analysis software.
